# Childhood trauma as a predictor of social cognition disturbances across psychosis spectrum: Data from the PREGAP Study

**DOI:** 10.1192/j.eurpsy.2024.80

**Published:** 2024-08-27

**Authors:** C. Aymerich, A. Catalan

**Affiliations:** ^1^Psychiatry, Basurto University Hospital; ^2^Psychiatry, Biobizkaia Health Research Institute, Bilbao; ^3^Neurosciences, Basque Country University (UPV/EHU), Leioa; ^4^GCV22/1/SAM, CIBERSAM, Madrid, Spain

## Abstract

**Introduction:**

Childhood trauma is a severe form of stress that has been strongly related to both the appearance of a psychotic disorder and the existance of social cognition disturbances. We hereby hypothesize childhood trauma might be a transdiagnostic marker of social cognition disturbances across the psychosis spectrum, regardless of the main diagnosis.

**Objectives:**

To investigate the effect of different forms of childhood trauma in social cognition impairments in first-episode psychosis, at-risk mental states for psychosis and healthy controls.

**Methods:**

Using cross-sectional data, we will examine the relationship between different kinds of chidlhood trauma (measured with the Childhood Trauma Questionnaire, CTQ) and several social cognition domains, including facial emotion recognition, theory of mind (assessed using the Movie Assessment for Social Cognition, MASC, The Hinting Task, and the Faux-Pas Questionnaire). Intra and inter-group differences be studied for three study groups, including patients with first-episode psychosis (n=60), subjects with at-risk mental states for psychosis (n=60), and healthy controls (n=60).

**Disclosure of Interest:**

None Declared

